# Perturbations in common and distinct inflammatory pathways associated with morning and evening fatigue in outpatients receiving chemotherapy

**DOI:** 10.1002/cam4.5435

**Published:** 2022-11-14

**Authors:** Kord M. Kober, Carolyn Harris, Yvette P. Conley, Anand Dhruva, Vasuda Dokiparthi, Marilyn J. Hammer, Jon D. Levine, Kate Oppegaard, Steven Paul, Joosun Shin, Anatol Sucher, Fay Wright, Brian Yuen, Adam B. Olshen, Christine Miaskowski

**Affiliations:** ^1^ School of Nursing University of California San Francisco California USA; ^2^ Helen Diller Family Comprehensive Cancer Center University of California San Francisco California USA; ^3^ School of Nursing University of Pittsburg Pittsburg Pennsylvania USA; ^4^ School of Medicine University of California San Francisco California USA; ^5^ Dana‐Farber Cancer Institute Boston Massachusetts USA; ^6^ Rory Meyers College of Nursing, New York University New York New York USA

**Keywords:** cancer, chemotherapy, cytokines, fatigue, gene expression, inflammation, knowledge network, pathway impact analysis

## Abstract

**Background:**

Moderate to severe fatigue occurs in up to 94% of patients with cancer. Recent evidence suggests that morning and evening fatigue are distinct dimensions of physical fatigue. The purposes of this study were to evaluate the transcriptome for common and distinct perturbed inflammatory pathways in patients receiving chemotherapy who reported low versus high levels of morning or low versus high levels of evening cancer‐related fatigue.

**Methods:**

Patients completed questionnaires during the week prior to their chemotherapy treatment. Severity of morning and evening fatigue was evaluated using the Lee Fatigue Scale. Gene expression and pathway impact analyses (PIA) were performed in two independent samples using RNA‐sequencing (*n* = 357) and microarray (*n* = 360). Patterns of interactions between and among these perturbed pathways were evaluated using a knowledge network (KN).

**Results:**

Across the PIA, nine perturbed pathways (FDR < 0.025) were common to both morning and evening fatigue, six were distinct for morning fatigue, and four were distinct for evening fatigue. KN (19 nodes, 39 edges) identified the phosphatidylinositol 3‐kinase (PI3K)‐Akt pathway node (perturbed in evening fatigue) with the highest betweenness (0.255) and closeness (0.255) centrality indices. The next highest betweenness centrality indices were seen in pathways perturbed in evening fatigue (i.e., nuclear factor kappa B: 0.200, natural killer cell‐mediated cytotoxicity: 0.178, mitogen‐activated protein kinase: 0.175).

**Conclusions:**

This study describes perturbations in common and distinct inflammatory pathways associated with morning and/or evening fatigue. PI3K‐Akt was identified as a bottleneck pathway. The analysis identified potential targets for therapeutic interventions for this common and devastating clinical problem.

## Graphical abstract

This study describes perturbations in common and distinct inflammatory pathways associated with morning and/or evening fatigue associated with clinically meaningful levels of morning and evening fatigue severity in oncology patients receiving chemotherapy. A knowledge network represents the pathway‐to‐pathway interconnections of common and distinct perturbed inflammatory pathways. The distinct pathways for morning (blue nodes) and evening (red nodes) fatigue group together within the knowledge network. The remaining interconnections between the two symptoms are common perturbed pathways (green nodes). PI3K‐Akt was identified as a bottleneck pathway. The analysis identified potential targets for therapeutic interventions for this common and devastating clinical problem.

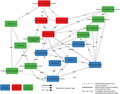

## 1 INTRODUCTION

Cancer‐related fatigue (CRF) exhibits a large amount of interindividual variability^1^ and has a negative impact on patients' ability to tolerate chemotherapy.^2^ Recent evidence suggests that morning and evening fatigue are distinct dimensions of physical fatigue.^3^ A major impediment to the development of effective treatments is the lack of knowledge of their underlying mechanism(s).^1^

While the mechanisms that underlie CRF are hypothesized to be multi‐factorial,^1^ the majority of the evidence supports inflammatory mechanisms. Although most of the studies focused on serum cytokines, an equally valid approach is to examine associations between low versus high levels of CRF and differences in gene RNA expression and perturbations in inflammatory pathways. Other than our work,^4^, ^5^, ^6^ only six studies, in four independent patient samples, have evaluated for associations between CRF and changes in gene expression.^7^, ^8^, ^9^, ^10^, ^11^, ^12^ In one study,^8^ the analysis was limited to pathways involved in mitochondrial function. In another study,^7^ only adrenergic, monoamine, and peptidergic pathways were evaluated. In the five studies that generated whole transcriptome data, three specifically targeted genes and pathways related to mitochondrial function^12^ and/or inflammation and immune function.^9^, ^10^, ^12^ Only two studies performed discovery analyses and reported findings on the whole transcriptome.^4^, ^11^ In our studies,^4^, ^5^, ^6^ perturbations in pathways involved in inflammation, neurotransmitter regulation, circadian rhythms, renal function, and energy metabolism were associated with evening fatigue severity in patients undergoing chemotherapy. Because mean fatigue scores were used in previous studies,^7^, ^8^, ^9^, ^10^, ^11^, ^12^ potential differences in the mechanisms that underlie diurnal variations in CRF were not evaluated. Therefore, the purposes of this study were to evaluate the transcriptome for perturbed inflammatory pathways in patients receiving chemotherapy who reported low versus high levels of morning or low versus high levels of evening CRF, evaluate for common and distinct perturbed inflammatory pathways between morning and evening CRF, and evaluate for patterns of interactions between and among these perturbed pathways using a knowledge network.^13^

## 2 MATERIALS AND METHODS

### 2.1 Patients and settings

This analysis is part of a larger study of oncology patients' symptom experiences.^14^ Eligible patients were ≥18 years of age; had a diagnosis of breast, gastrointestinal, gynecological, or lung cancer; had received chemotherapy within the preceding 4 weeks; were scheduled to receive at least two additional cycles of chemotherapy; were able to read, write, and understand English; and gave written informed consent. Patients were recruited from two Comprehensive Cancer Centers, one Veteran's Affairs hospital, and four community‐based oncology programs.

### 2.2 Study procedures

The study was approved by the Institutional Review Board at each of the study sites. Of the 2234 patients approached, 1343 consented to participate. A major reason for refusal was being overwhelmed with their cancer treatment. Eligible patients were approached in the infusion unit during their first or second cycle of chemotherapy by a member of the research team to discuss participation and obtain written informed consent. Blood was collected at the enrollment assessment.

### 2.3 Instruments

#### 2.3.1 Phenotypic characteristics

Patients completed a demographic questionnaire, Karnofsky Performance Status (KPS) scale,^15^ Self‐Administered Comorbidity Questionnaire (SCQ),^16^ and Alcohol Use Disorders Identification Test (AUDIT).^17^ Toxicity and emetogenicity of the chemotherapy regimen were rated using the MAX2 index^18^ and published guidelines,^19^ respectively. Medical records were reviewed for disease and treatment information.

#### 2.3.2 Lee Fatigue Scale (LFS)

The 18‐item LFS was used to assess physical fatigue and energy.^20^ Each item was rated on a 0 to 10 numeric rating scale. Mean scores were calculated for the 13 fatigue items. Higher scores indicate greater fatigue severity. Using separate LFS questionnaires, patients rated each item based on how they felt within 30 minutes of awakening (i.e., morning fatigue) and prior to going to bed (i.e., evening fatigue).

### 2.4 Data analyses

#### 2.4.1 Patient samples

Of the 717 patients who provided a blood sample, 357 were processed using RNA‐sequencing (i.e., RNA‐seq sample) and 360 were processed using microarrays (i.e., microarray sample).

#### 2.4.2 Imputation process

Missing data for demographic and clinical characteristics were imputed by the *k*‐nearest‐neighbors method, with *k* = 9. For continuous variables, the Euclidean distance was used to find the nearest neighbors. The imputed value was the weighted average of the nearest neighbors, with each weight originally exp(−dist[*x*,*j*]), after which the weights were scaled to one. For categorical variables, the distance was 0 if the predictor and the neighbor had the same value and 1 if they did not and the imputed value was the mode of the nearest neighbors.

#### 2.4.3 Phenotypic data

Data from the two samples were analyzed separately using R (version 4.1, https://www.R‐project.org/). Patients were classified into low (<3.2) and high (≥3.2) morning fatigue and low (<5.6) and high (≥5.6) evening fatigue groups using clinically meaningful cutoff scores.^21^ For each sample, differences in demographic and clinical characteristics between the fatigue groups were evaluated using parametric and nonparametric tests. Significant characteristics (*p*‐value of <0.05) were entered into a logistic regression analysis to adjust for covariates in the gene expression analyses. For the final model, variables were selected using a backward stepwise logistic regression approach based on the likelihood ratio test. The area under the curve of the receiver operating characteristic curve was used to evaluate the overall adequacy of the regression models.^22^

#### 2.4.4 Acquisition and processing of gene expression data

Gene expression analyses are described in detail elsewhere.^14^ In brief, total RNA isolated from peripheral blood was quantified for 357 patients using RNA‐seq and 360 patients using the HumanHT‐12 v4.0 Expression BeadChip (Illumina, San Diego, CA) microarray.

#### 2.4.5 Differential expression, pathway impact analyses (PIA), and knowledge network construction

For morning and evening fatigue, differential expression was quantified using empirical Bayes models using edgeR^43^ for the RNA‐seq sample and limma^44^ for the microarray sample.^14^ These analyses were adjusted for phenotypic characteristics retained in the final logistic models. The models included surrogate variables not associated with morning or evening fatigue to adjust for variation due to unmeasured sources.^23^ Only genes with a common direction of expression across the two samples were retained for subsequent analyses.

PIA was used to interpret the gene expression results in the context of CRF‐related mechanisms.^14^ PIA included the results of the differential expression analyses for all genes (i.e., cutoff free) to determine the probability of pathway perturbations using Pathway Express.^24^ A total of 225 signaling pathways were identified using the Kyoto Encyclopedia of Genes and Genomes (KEGG) database.^25^ For each sample, a separate test was performed for each pathway. Then, Fisher's Combined Probability method was used to combine these test results to obtain a single test (global) of the null hypothesis.^26^ Significance of the combined transcriptome‐wide PIA was assessed using a strict false discovery rate (FDR) of 2.5 × 10^−3^ under the Benjamini‐Hochberg procedure.^27^ Then, these results were evaluated for common and distinct inflammatory pathways associated with morning and evening fatigue.

Finally, an unweighted knowledge network was created based on interconnections among these inflammatory pathways using KEGG pathway maps. A knowledge network is a multi‐edge graph that combines heterogeneous information from several sources, provides information about the nature and degree of interactions between/among nodes, and allows for the identification of nodes that have structural importance.^13^ Nodes were defined as perturbed inflammatory KEGG signaling pathways identified in our analyses. Edges were defined from the KEGG pathway map images. Edges were categorized as being: identified in KEGG as an indirect link or unknown reaction, identified in KEGG shared members (i.e., genes or their products), or identified by authors as shared members. To gain insights into the structural importance of each node, two centrality indices (i.e., closeness and betweenness) were estimated. The number of connected nodes (NCN) was calculated as the number of unique node‐to‐node connections. The proportion of NCNs was calculated as the NCN divided by the average NCN across all nodes. Summary statistics were calculated for the final network using Cytoscape.^28^

## 3 RESULTS

### 3.1 RNA‐seq performance

Of the 357 patients in the RNA‐seq sample, 350 had morning fatigue data (Figures S1 and S2). Of these patients, one was excluded for poor RNA quantification. The median library threshold size was 8,995,065 reads. Following quality control filters, 10,574 genes were included in the differential expression analysis. Of these 357 patients in the RNA‐seq sample, 349 patients had evening fatigue data. Of these patients, one was excluded for poor RNA quantification. The median library size was 9,013,020 reads. Following quality control filters, 10,612 genes were included in the differential expression analysis.

### 3.2 Microarray performance

Of the 360 patients in the microarray sample, 353 had morning fatigue data (Figures S1 and S2). Of these patients, four were excluded due to poor RNA quantification. Following quality control filters, 44,555 loci were included in the differential expression analysis. Of these 360 patients, 352 had evening fatigue data. Of these patients, four were excluded due to poor RNA quantification. Following quality control filters, 44,551 probes were included in the differential expression analysis.

### 3.3 Logistic regression analyses of phenotypic characteristics

Details on differences in phenotypic characteristics between morning and evening fatigue groups for each sample are provided in Tables S1–S4. In terms of morning fatigue, for the RNA‐seq sample, 11 characteristics and for the microarray sample, seven characteristics were retained in the final logistic regression models and were used as covariates in the gene expression analyses, respectively (Table 1). In terms of evening fatigue, for the RNA‐seq sample, five characteristics and for the microarray sample, three variables were retained in the final logistic regression models and were used as covariates in the gene expression analyses (Table 2).

**TABLE 1:** Multiple logistic regression analyses predicting high morning fatigue group membership

RNA‐sequencing sample (*n* = 349; 46.1% Low and 53.9% High Morning Fatigue)			
Predictors	Odds ratio	95% CI	*p*‐value
Age	0.94	0.91, 0.96	<0.001
Body mass index (kg/m^2^)	1.05	1.00, 1.11	0.067
Karnofsky Performance Status score	0.96	0.94, 0.98	<0.001
Number of comorbidities	0.66	0.38, 1.12	0.128
Self‐Administered Comorbidity Questionnaire score	1.36	1.08, 1.75	0.012
Time since diagnosis (years)	1.12	1.01, 1.26	0.045
Gender (female)	2.41	1.10, 5.40	0.030
Lives alone (yes)	3.06	1.60, 6.03	<0.001
Self‐reported diagnosis of depression (yes)	1.85	0.86, 4.06	0.119
Cancer diagnosis			
Breast	1.00		
Gastrointestinal	0.82	0.40, 1.68	0.590
Gynecological	0.61	0.27, 1.36	0.228
Lung	3.06	1.04, 9.59	0.005
Type of prior cancer treatment			
No prior treatment	1.00		
Only surgery, CTX, or RT	1.98	1.05, 3.78	0.036
Surgery and CTX, or surgery and RT, or CTX and RT	1.20	0.52, 2.78	0.672
Surgery and CTX and RT	2.53	0.82, 8.05	0.109
Overall model fit: AUC of the ROC = 0.825			
Microarray sample (n = 349; 53.3% Low and 46.7% High Morning Fatigue)			
Predictors	Odds Ratio	95% CI	p‐value
Age (years)	0.97	0.95, 0.99	0.005
Karnofsky Performance Status score	0.97	0.95, 0.99	0.007
Self‐Administered Comorbidity Questionnaire score	1.07	0.98, 1.17	0.150
Married/partnered (yes)	0.67	0.41, 1.10	0.113
Exercise on a regular basis (yes)	0.54	0.33, 0.91	0.196
Self‐reported diagnosis of depression (yes)	1.70	0.91, 3.17	0.094
Antiemetic regimen			
None	1.00		
Steroid alone or serotonin receptor antagonist alone	1.12	0.48, 2.65	0.796
Serotonin receptor antagonists and steroid	0.77	0.35, 1.71	0.516
NK‐1 receptor antagonist and two other antiemetics	1.77	0.73, 4.33	0.209
Overall model fit: AUC of the ROC = 0.724			

Abbreviations: AUC, area under curve; CI, confidence interval; CTX, chemotherapy; ROC, receiver operating characteristic; RT, radiation therapy.

**TABLE 2:** Multiple logistic regression analyses predicting high evening fatigue group membership

RNA‐sequencing Sample (*n* = 348; 45.1% Low and 54.9% High Evening Fatigue)			
Predictors	Odds Ratio	95% CI	*p*‐value
Age (years)	0.97	0.96, 0.99	0.006
Karnofsky Performance Status score	0.97	0.95, 0.99	0.001
Gender (female)	2.85	1.63, 5.08	<0.001
Born prematurely (yes)	2.83	0.86, 12.8	0.117
Type of prior cancer treatment			
No prior treatment	1.00		
Only surgery, CTX, or RT	2.07	1.19, 3.66	0.011
Surgery and CTX, or surgery and RT, or CTX and RT	1.82	0.91, 3.67	0.091
Surgery and CTX and RT	2.60	1.17, 5.97	0.021
Overall model fit: AUC of the ROC = 0.722			
Microarray sample (*n* = 348; 47.7% Low and 52.3% High Evening Fatigue			
Predictors	Odds ratio	95% CI	*p*‐value
Body mass index (kg/m^2)^	1.04	1.01, 1.08	0.022
Karnofsky Performance Status score	0.97	0.95, 0.99	0.002
Childcare responsibilities (yes)	1.83	1.10, 3.09	0.022
Overall model fit: AUC of the ROC = 0.644			

Abbreviations: AUC, area under curve; CI, confidence interval; CTX, chemotherapy; ROC, receiver operating characteristic; RT, radiation therapy.

### 3.4 Perturbed signaling pathways

**FIGURE 1:**
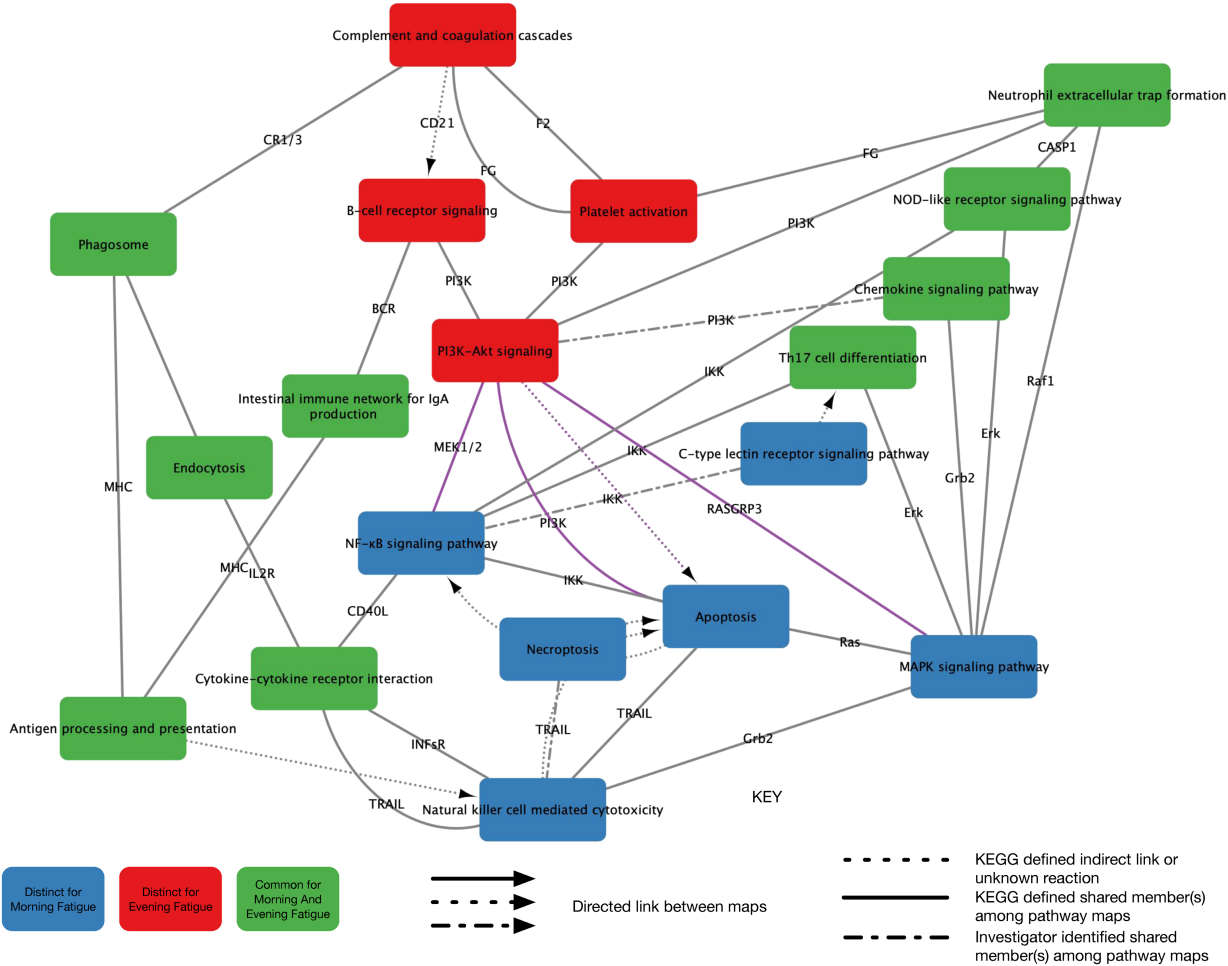
A knowledge network generated from connections among the common and distinct perturbed inflammation‐related KEGG signaling pathways associated with morning and evening cancer‐related fatigue. Nodes represent each of the KEGG signaling pathways. Edges represent connections between the pathways. Edge labels identify a shared member of the network. Edges between pathways unique to morning and evening fatigue are colored purple.

Of the 225 KEGG pathways identified, 221 had sufficient data for evaluation across all four analyses. In terms of morning fatigue, 69 pathways were significantly perturbed and 15 were related to inflammatory mechanisms. In terms of evening fatigue, 54 pathways were significantly perturbed and 13 were related to inflammatory mechanisms. Across the PIA, nine perturbed pathways were common to both morning and evening fatigue, six were distinct for morning fatigue, and four were distinct for evening fatigue (Table 3, Figure 1).

**TABLE 3:** Common and distinct perturbed inflammatory KEGG pathways for morning and evening fatigue

Pathway ID	Pathway name	Statistics morning fatigue	Statistics evening fatigue
Both morning and evening fatigue			
hsa04612	Antigen processing and presentation	*X* ^2^ = 30.41, *p* = 2.85 × 10^−4^	*X* ^2^ = 24.52, *p* = 6.05 × 10^−4^
hsa04144	Endocytosis	*X* ^2^ = 28.21, *p* = 3.57 × 10^−4^	*X* ^2^ = 30.41, *p* = 2.98 × 10^−4^
hsa04145	Phagosome	*X* ^2^ = 26.51, *p* = 4.59 × 10^−4^	*X* ^2^ = 27.19, *p* = 3.67 × 10^−4^
hsa04060	Cytokine‐cytokine receptor interaction	*X* ^2^ = 26.01, *p* = 4.77 × 10^−4^	*X* ^2^ = 26.01, *p* = 4.09 × 10^−4^
hsa04613	Neutrophil extracellular trap formation	*X* ^2^ = 25.13, *p* = 4.77 × 10^−4^	*X* ^2^ = 20.88, *p* = 2.08 × 10^−3^
hsa04062	Chemokine signaling pathway	*X* ^2^ = 24.41, *p* = 4.77 × 10^−4^	*X* ^2^ = 21.30, *p* = 1.56 × 10^−3^
hsa04659	Th17 cell differentiation	*X* ^2^ = 23.41, *p* = 6.61 × 10^−4^	*X* ^2^ = 22.47, *p* = 1.10 × 10^−3^
hsa04621	NOD‐like receptor signaling pathway	*X* ^2^ = 23.03, *p* = 7.05 × 10^−4^	*X* ^2^ = 27.63, *p* = 3.63 × 10^−4^
hsa04672	Intestinal immune network for IgA production	*X* ^2^ = 22.93, *p* = 7.05 × 10^−4^	*X* ^2^ = 26.01, *p* = 4.09 × 10^−4^
**Only morning fatigue**			
hsa04217	Necroptosis	*X* ^2^ = 24.32, *p* = 4.77 × 10^−4^	*X* ^2^ = 18.44, *p* = 3.66 × 10^−3^
hsa04625	C‐type lectin receptor signaling pathway	*X* ^2^ = 23.35, *p* = 6.61 × 10^−4^	*X* ^2^ = 14.70, *p* = 1.12 × 10^−2^
hsa04010	MAPK signaling pathway	*X* ^2^ = 22.66, *p* = 7.60 × 10^−4^	*X* ^2^ = 19.21, *p* = 2.77 × 10^−3^
hsa04650	Natural killer cell‐mediated cytotoxicity	*X* ^2^ = 20.67, *p* = 1.48 × 10^−3^	*X* ^2^ = 18.03, *p* = 3.97 × 10^−3^
hsa04064	NF‐κB signaling pathway	*X* ^2^ = 20.06, *p* = 1.85 × 10^−3^	*X* ^2^ = 18.01, *p* = 3.97 × 10^−3^
hsa04210	Apoptosis	*X* ^2^ = 19.07, *p* = 2.44 × 10^−3^	*X* ^2^ = 16.59, *p* = 6.18 × 10^−3^
**Only evening fatigue**			
hsa04610	Complement and coagulation cascades	*X* ^2^ = 17.27, *p* = 4.21 × 10^−3^	*X* ^2^ = 20.58, *p* = 1.80 × 10^−3^
hsa04611	Platelet activation	*X* ^2^ = 13.63, *p* = 1.39 × 10^−2^	*X* ^2^ = 24.05, *p* = 7.19 × 10^−4^
hsa04151	PI3K‐Akt signaling pathway	*X* ^2^ = 13.25, *p* = 1.58 × 10^−2^	*X* ^2^ = 19.66, *p* = 2.39 × 10^−3^
hsa04662	B‐cell receptor signaling pathway	*X* ^2^ = 12.30, *p* = 2.22 × 10^−2^	*X* ^2^ = 20.02, *p* = 2.10 × 10^−3^

Abbreviations: AKT, Protein kinase B; KEGG, Kyoto Encyclopedia of Genes and Genomes; MAPK, mitogen‐activated protein kinase; NF‐κB, nuclear factor kappa‐light‐chain‐enhancer of activated B cells; NOD, nucleotide‐binding and oligomerization domain; PI3K, Phosphoinositide 3‐kinases; *p*, global perturbation *p*‐value adjusted using the Benjamini‐Hochberg procedure (pPert <2.50 × 10^−3^ was considered significantly perturbed); Th17, T‐helper 17.

### 3.5 Knowledge network

The knowledge network consists of 19 nodes with 39 edges (average number of neighbors = 3.32, Figure 1). The phosphatidylinositol 3‐kinase (PI3K)‐Akt pathway node was the most interconnected (2.11 times the average) and had the highest betweenness (0.255) and closeness (0.255) centrality indices (Table 4). The three pathways with the second highest number of interconnections (1.51 times the average) had the next highest betweenness centrality indices (i.e., nuclear factor kappa B (NF‐κB) signaling: 0.200, natural killer (NK) cell‐mediated cytotoxicity: 0.178, mitogen‐activated protein kinase (MAPK) signaling: 0.175).

**TABLE 4:** Summary statistics for the perturbed inflammatory KEGG signaling pathway fatigue knowledge network

Pathway ID	Perturbed Symptom	Pathway Name	BC*	CC	NCN	NCN Ratio
hsa04151	PM	PI3K‐Akt signaling	0.255	0.563	7	2.11
hsa04064	AM	NF‐KB signaling pathway	0.200	0.529	5	1.51
hsa04650	AM	Natural killer cell‐mediated cytotoxicity	0.178	0.514	5	1.51
hsa04010	AM	MAPK signaling pathway	0.175	0.545	5	1.51
hsa04060	Both	Cytokine‐cytokine receptor interaction	0.100	0.462	2	0.60
hsa04662	PM	B‐cell receptor signaling	0.092	0.450	3	0.90
hsa04612	Both	Antigen processing and presentation	0.088	0.429	3	0.90
hsa04210	AM	Apoptosis	0.080	0.514	4	1.21
hsa04145	Both	Phagosome	0.063	0.383	3	0.90
hsa0610	PM	Complement and coagulation cascades	0.060	0.391	3	0.90
hsa04611	PM	Platelet activation	0.056	0.439	3	0.90
hsa04613	Both	Neutrophil extracellular trap formation	0.032	0.450	4	1.21
hsa04144	Both	Endocytosis	0.032	0.375	2	0.60
hsa04659	Both	Th17 cell differentiation	0.018	0.409	3	0.90
hsa04672	Both	Intestinal immune network for IgA production	0.011	0.367	2	0.60
hsa04621	Both	NOD‐like receptor signaling pathway	0.011	0.429	3	0.90
hsa04062	Both	Chemokine signaling pathway	0.000	0.409	2	0.60
hsa04625	AM	C‐type lectin receptor signaling pathway	0.000	0.367	2	0.60
hsa04217	AM	Necroptosis	0.000	0.391	2	0.60

Abbreviations: AKT, Protein kinase B; KEGG, Kyoto Encyclopedia of Genes and Genomes; BC, Betweenness Centrality; CC, Closeness Centrality; MAPK, mitogen‐activated protein kinase; NF‐κB, nuclear factor kappa‐light‐chain enhancer of activated B cells; NCN, Number of connected nodes; NCN ratio, pathway NCN/average NCN; NOD, nucleotide‐binding and oligomerization domain; PI3K, phosphoinositide 3‐kinases; Th17, T‐helper 17.

^a^ Table organized in descending order of BC.

## 4 DISCUSSION

This study is the first to identify common and distinct perturbed inflammatory pathways associated with clinically meaningful levels of morning and evening fatigue severity in oncology patients receiving chemotherapy. These findings are congruent with our hypothesis that morning and evening fatigue are distinct but related symptoms.^3^ As shown in Figure 1, the distinct pathways for the morning (blue nodes) and evening (red nodes) fatigue group together within the knowledge network. Only the PI3K‐Akt signaling pathway that is distinct from evening fatigue provides a direct connection to the perturbed pathways for morning fatigue. All of the other interconnections between the two symptoms are common perturbed pathways (green nodes). Most of the common pathways (e.g., antigen processing and presentation, chemokine signaling pathway, cytokine‐cytokine receptor interaction) were identified in our previous studies of evening fatigue^4^, ^5^, ^6^ and in studies of average fatigue^8^, ^10^, ^11^ and will not be discussed in detail. The remainder of this discussion focuses on the more novel common and distinct inflammatory pathways.

### 4.1 Common pathways for morning and evening fatigue

The new common pathway identified in this analysis is neutrophil extracellular trap (NET) formation. This pathway is involved in the creation and release of extracellular lattices of decondensed chromatin and granule proteins that contain circulating cell‐free DNA from neutrophils (i.e., NETosis).^29^ The NET formation pathway models the numerous processes that trigger NETosis by microorganisms, endogenous damage‐associated molecular patterns, and antibodies through signaling cascades and effector proteins.^30^ Dysregulation of NETs occurs in response to inflammation.^31^ Preclinical findings suggest that dysregulation of NETs is associated with muscle pain^32^ and joint pain.^33^ While no studies have reported on associations between CRF and the NET formation pathway, in a study of cancer survivors, those with chronic fatigue had higher neutrophil counts.^11^ Given That nets are hypothesized to influence sleep quality and physical activity,^34^, ^35^ and that an increase in the number of a subpopulation of neutrophils (i.e., low‐density granulocytes) is hypothesized to act as NET inducers,^36^ an examination of associations between CRF severity and changes in the composition and levels of subtypes of neutrophils is warranted.

### 4.2 Distinct pathways for morning fatigue

Of the six distinct pathways for morning fatigue, neither C‐type lectin receptor signaling nor necroptosis was identified in previous gene expression studies of CRF. Interestingly, both pathways are implicated in the activation of the NF‐κb signaling pathway and associated inflammatory responses.^37^, ^38^ C‐type lectin receptors (clrs) are a type of pattern recognition receptor that induces diverse innate immune responses expressed in dendritic cells.^38^ Dendritic cells act as messengers between the adaptive and innate immune systems through interactions with T and B cells that are located in the intestine, stomach, and lung.^39^ clrs can activate NF‐κb signaling (morning fatigue) and pathways for T‐cell differentiation (both morning and evening fatigue). While not evaluated in patients undergoing chemotherapy, in a study of patients with chronic fatigue syndrome/myalgic encephalomyelitis (CFS/ME),^40^ the C‐type lectin receptor signaling pathway was a member of a cluster of pathways enriched for differentially methylated genes associated with CFS severity. CRF and CFS/ME share gene expression patterns at a subset of genes^7^ and may share underlying mechanisms.^41^

The necroptosis pathway models a programmed form of cell death induced by the binding of a specific set of death receptors (e.g., tumor necrosis factor [TNF] receptor 1 [TNFR1] and TNF‐related apoptosis‐inducing ligand receptor [TRAILR]).^42^ Necroptosis promotes the activation of the NF‐κb signaling pathway and induction of cytokine expression.^37^ While not evaluated in patients undergoing chemotherapy, in a study of men with prostate cancer,^43^ compared to nonfatigued controls, TRAIL cytokine expression and mrna expression of TRAILR were upregulated in fatigued patients.

The four remaining pathways that are distinct for morning fatigue (i.e., MAPK signaling,^4^ NK cell‐mediated cytotoxicity,^4^, ^5^ apoptosis,^4^ and NF‐κb signaling^5^, ^10^) were reported previously. The shared interconnections among these pathways represent common inflammatory mechanisms. Interestingly, in our knowledge network, the PI3K‐Akt pathway (discussed in the next section) is the only pathway that provides direct interconnections between the distinct pathways for morning fatigue (i.e., MAPK signaling, apoptosis, and NF‐κb signaling).

### 4.3 Distinct pathways for evening fatigue

Of the four distinct pathways for evening fatigue, PI3K‐Akt signaling and platelet activation were not identified in previous gene expression studies. The PI3K‐Akt pathway has the highest betweenness centrality index and shares edges with the three pathways with the next highest betweenness centrality indices (i.e., MAPK signaling, apoptosis, and NF‐κb signaling). The betweenness centrality index identifies “bottleneck” nodes that form bridges so that two communities (i.e., morning and evening pathways) can communicate with each other and can play a key role in the modularization of a network.^13^ As a “bottleneck” node, the PI3K‐Akt pathway may influence the inflammatory mechanisms for both morning and evening fatigue. The PI3K‐Akt pathway integrates receptor‐mediated signaling with cell metabolism and can suppress coagulation and inflammation.^44^ In addition, it responds to cytokines, growth factors, and hormones^45^ and can activate the NF‐kb signaling^46^ and apoptosis^45^ pathways.

Given that the PI3K/AKT pathway plays a significant role in oncogenesis,^47^ and that a common side effect associated with the administration of PI3K pathway inhibitors is fatigue,^48^ our findings suggest that this dysregulation of this pathway, either by cancer or its treatment, may be integral to the development and/or maintenance of CRF. In addition, in a preclinical study of postoperative fatigue,^49^ the administration of Ginsenoside Rb1 (a compound isolated from ginseng) had a positive effect. The authors suggested that this antifatigue effect occurred through the activation of the PI3K/Akt pathway. This pathway requires additional investigation to elucidate how patterns of dysregulation associated with cancer itself and/or various treatments influence the development and/or maintenance of CRF.

Platelet activation is involved in hemostasis, thrombosis, and intercellular communication that mediate inflammatory and immunomodulatory mechanisms.^50^ While no studies have identified an association between CRF and this pathway, CFS is hypothesized to be associated with a hypercoagulable state.^51^

### 4.4 Strengths and limitations

While this study had a relatively large sample size, had well‐phenotyped patients, included rigorous quality controls, set strict criteria for differential expression and pathway perturbation selection, and provided results from independent tests across two samples, some limitations warrant consideration. First, given that this evaluation is the first to report on associations between morning fatigue and gene expression changes, these findings warrant confirmation. Second, because morning and evening fatigue were assessed during chemotherapy, future studies need to examine associations between CRF from other types of cancers and cancer treatments and various pathway perturbations. Longitudinal studies are needed that assess for associations between changes in CRF and changes in gene expression and pathway perturbations. Given the heterogeneity of CRF instruments,^52^, ^53^ and that no gold standard exists for the assessment of CRF,^54^ future studies need to evaluate whether the same perturbed pathways are identified using a variety of instruments. The strict FDR cutoff excluded some pathways on either side of the cutoff. Therefore, findings need to be interpreted as highlighting relative versus absolute differences in pathway perturbations between morning and evening fatigue.

## 5 CONCLUSIONS

This study is the first to describe perturbations in common and distinct inflammatory pathways associated with morning and/or evening fatigue and to use a knowledge network approach to identify pathway‐to‐pathway interactions. Our findings suggest the PI3K‐Akt signaling pathway is a “bottleneck” pathway. Given that some of the signaling pathways are cell‐type specific, future research should evaluate within and across specific immune cells for differences in expression and epigenetic regulatory processes. Finally, given the strengths of our findings and the evidence that suggests that synergistic interactions occur between inflammatory and neuroendocrine processes,^1^, ^55^ future research needs to examine these pathways.

## AUTHOR CONTRIBUTIONS

**Kord M. Kober:** Conceptualization (lead); data curation (equal); formal analysis (equal); funding acquisition (lead); investigation (lead); methodology (lead); project administration (lead); resources (equal); software (equal); visualization (equal); writing – original draft (lead); writing – review and editing (lead). **Carolyn Harris:** Conceptualization (supporting); formal analysis (supporting); methodology (supporting); writing – review and editing (equal). **Yvette P Conley:** Conceptualization (supporting); methodology (supporting); writing – review and editing (equal). **Anand Dhruva:** Methodology (supporting); writing – review and editing (equal). **Vasuda Dokiparthi:** Formal analysis (equal); writing – review and editing (equal). **Marilyn J Hammer:** Methodology (supporting); writing – review and editing (equal). **JD Levine:** Conceptualization (supporting); methodology (supporting); writing – review and editing (equal). **Kate Oppegaard:** Writing – review and editing (equal). **Steven M Paul:** Formal analysis (supporting); writing – review and editing (equal). **Joosun Shin:** Writing – review and editing (equal). **Anatol Sucher:** Investigation (supporting); methodology (supporting); writing – review and editing (equal). **Fay Wright:** Conceptualization (supporting); writing – review and editing (equal). **Brian Yuen:** Investigation (supporting); writing – review and editing (equal). **Adam Olshen:** Conceptualization (supporting); formal analysis (supporting); methodology (equal); writing – review and editing (equal). **Christine Miaskowski:** Conceptualization (equal); funding acquisition (equal); methodology (equal); resources (equal); writing – original draft (equal); writing – review and editing (equal).

## FUNDING INFORMATION

This study was funded by grants from the American Cancer Society and the National Institutes of Health/National Cancer Institute (CA134900, CA233774, and CA082103). Dr. Miaskowski is an American Cancer Society Clinical Research Professor.

## CONFLICT OF INTEREST

The authors declare that they have no conflict of interest.

## Supporting information


Figure S1.

Figure S2.

Table S1.

Table S2.

Table S3.

Table S4.

## Data Availability

The data that support the findings of this study are available from the corresponding author upon reasonable request.
