# Supplementary material for: Perturbations in common and distinct inflammatory pathways associated with morning and evening fatigue in outpatients receiving chemotherapy
Source: Cancer Med. 2022 Nov 14;12(6):7369–80. doi: 10.1002/cam4.5435 (PMC10067125; doi:10.1002/cam4.5435)
Supplement: Supplementary file 1 — Figure S1. [file CAM4-12-7369-s002.docx]

Supplementary Figure 1: Flow diagram of the number of patients available for phenotypic, gene

expression (GE), and pilot methylation (MT) analyses of morning fatigue (MF).
