# Supplementary material for: Perturbations in common and distinct inflammatory pathways associated with morning and evening fatigue in outpatients receiving chemotherapy
Source: Cancer Med. 2022 Nov 14;12(6):7369–80. doi: 10.1002/cam4.5435 (PMC10067125; doi:10.1002/cam4.5435)
Supplement: Supplementary file 2 — Figure S2. [file CAM4-12-7369-s005.docx]

Supplementary Figure 2: Flow diagram of the number of patients available for phenotypic and gene

expression (GE) analyses of evening fatigue (EF).
