# Supplementary material for: Perturbations in common and distinct inflammatory pathways associated with morning and evening fatigue in outpatients receiving chemotherapy
Source: Cancer Med. 2022 Nov 14;12(6):7369–80. doi: 10.1002/cam4.5435 (PMC10067125; doi:10.1002/cam4.5435)
Supplement: Supplementary file 3 — Table S1. [file CAM4-12-7369-s004.docx]

Supplemental Table 1. Differences in Demographic and Clinical Characteristics Between Patients in the RNA-Seq Sample with Low and High Morning Fatigue

| Characteristic | Low Morning Fatigue  46.1% (n=161) | High Morning Fatigue  53.9% (n=188) | Statistics |
| --- | --- | --- | --- |
|  | Mean (SD) | Mean (SD) |  |
| Age (years) | 59.5 (11.4) | 53.6 (12.5) | t = 4.56, p < 0.001 |
| Education (years) | 16.2 (3.2) | 15.9 (2.9) | t = 1.04, p = 0.300 |
| Body mass index (kg/m^2^) | 25.4 (5.0) | 26.6 (6.0) | t = -2.02, p = 0.044 |
| KPS score | 81.6 (11.8) | 74.0 (11.8) | t = 5.98, p < 0.001 |
| Number of comorbidities | 2.3 (1.4) | 2.8 (1.6) | t = -3.03, p = 0.003 |
| SCQ score | 4.9 (3.0) | 6.6 (3.8) | t = -4.54, p < 0.001 |
| AUDIT score | 2.8 (1.8) | 2.8 (2.2) | t = -0.16, p = 0.872 |
| Time since diagnosis (years) | 1.3 (2.4) | 2.0 (3.6) | U, p = 0.031 |
| Time since diagnosis (years, median) | 0.41 | 0.45 |  |
| Number of prior cancer treatments | 1.3 (1.3) | 1.7 (1.5) | t = -2.63, p = 0.009 |
| Number of metastatic sites including lymph node involvement | 1.2 (1.1) | 1.3 (1.3) | t = -0.67, p = 0.502 |
| Number of metastatic sites excluding lymph node involvement | 0.71 (0.94) | 0.79 (1.1) | t = -0.66, p = 0.508 |
| MAX2 score | 0.18 (0.08) | 0.18 (0.08) | t = -0.97, p = 0.333 |
| Hemoglobin (g/dL) | 11.5 (1.4) | 11.4 (1.4) | t = 0.68, p = 0.495 |
| Hematocrit (%) | 34.6 (4.0) | 34.2 (4.1) | t = 0.99, p = 0.324 |
|  | % (n) | % (n) |  |
| Gender  Female  Male | 70.2 (113)  29.8 (48) | 85.1 (160)  14.9 (28) | FE, p = 0.001 |
| Ethnicity  White  Black  Asian or Pacific Islander  Hispanic mixed or other | 64.6 (104)  8.1 (13)  16.8 (27)  10.6 (17) | 63.3 (119)  7.4 (14)  16.0 (30)  13.3 (25) | X^2^ = 0.64, p = 0.887 |
| Married or partnered (% yes) | 70.2 (113) | 53.7 (101) | FE, p = 0.002 |
| Lives alone (% yes) | 14.9 (24) | 31.4 (59) | FE, p < 0.001 |
| Childcare responsibilities (% yes) | 19.9 (32) | 22.9 (43) | FE, p = 0.516 |
| Care of adult responsibilities (% yes) | 8.1 (13) | 6.9 (13) | FE, p = 0.689 |
| Born prematurely (% yes) | 3.1 (5) | 5.9 (11) | FE, p = 0.306 |
| Currently employed (% yes) | 35.4 (57) | 29.3 (55) | FE, p = 0.250 |
| Income  <$30,000  $30,000 to <$70,000  $70,000 to <$100,000  ≥$100,000 | 14.3 (23)  20.5 (33)  23.6 (38)  41.6 (67) | 26.6 (50)  20.2 (38)  18.6 (35)  34.6 (65) | U, p = 0.008 |
| Specific comorbidities (% yes)  Heart disease  High blood pressure  Lung disease  Diabetes  Ulcer or stomach disease  Kidney disease  Liver disease  Anemia or blood disease  Depression  Osteoarthritis  Back pain  Rheumatoid arthritis | 6.2 (10)  34.8 (56)  7.5 (12)  9.9 (16)  3.7 (6)  1.9 (3)  6.2 (10)  8.7 (14)  11.2 (18)  11.8 (19)  23.6 (38)  3.1 (5) | 6.4 (12)  30.3 (57)  11.2 (21)  13.8 (26)  5.3 (10)  0.5 (1)  6.9 (13)  12.2 (23)  30.3 (57)  13.3 (25)  41.0 (77)  5.3 (10) | FE, p = 1.000  FE, p = 0.422  FE, p = 0.274  FE, p = 0.323  FE, p = 0.610  FE, p = 0.339  FE, p = 0.832  FE, p = 0.301  FE, p < 0.001  FE, p = 0.747  FE, p < 0.001  FE, p = 0.429 |
| Exercise on a regular basis (% yes) | 70.8 (114) | 63.8 (120) | FE, p = 0.173 |
| Smoking current or history of (% yes) | 31.1 (50) | 38.3 (72) | FE, p = 0.177 |
| Cancer diagnosis  Breast  Gastrointestinal  Gynecological  Lung | 32.9 (53)  44.1 (71)  16.8 (27)  6.2 (10) | 44.7 (84)  26.6 (50)  15.4 (29)  13.3 (25) | X^2^ = 15.16, p = 0.002  0 < 1  0 > 1  NS  NS |
| Type of prior cancer treatment  No prior treatment  Only surgery, CTX, or RT  Surgery & CTX, or surgery & RT, or CTX & RT  Surgery & CTX & RT | 33.5 (54)  37.9 (61)  20.5 (33)  8.1 (13) | 22.9 (43)  44.7 (84)  15.4 (29)  17.0 (32) | X^2^ = 11.15, p = 0.011  No significant post hoc contrasts |
| CTX cycle length  14 day cycle  21 day cycle  28 day cycle | 50.9 (82)  42.9 (69)  6.2 (10) | 44.7 (84)  45.7 (86)  9.6 (18) | X^2^ = 2.10, p = 0.350 |
| Emetogenicity of CTX  Minimal/low  Moderate  High | 11.8 (19)  70.2 (113)  18.0 (29) | 19.7 (37)  57.4 (108)  22.9 (43) | X^2^ = 6.57, p = 0.037  No significant post hoc contrasts |
| Antiemetic regimens  None  Steroid alone or serotonin receptor antagonist alone  Serotonin receptor antagonist and steroid  NK-1 receptor antagonist and two other antiemetics | 5.6 (9)  14.9 (24)  54.0 (87)  25.5 (41) | 4.8 (9)  20.2 (38)  44.1 (83)  30.9 (58) | X^2^ = 4.11, p = 0.250 |
| LFS morning fatigue score at enrollment | 1.5 (0.9) | 5.5 (1.7) | t = -27.10, p < 0.001 |

Abbreviations: AUDIT = Alcohol Use Disorders Identification Test; CTX = chemotherapy; dl = deciliters; FE = Fisher's exact test; g = grams; kg = kilograms; KPS = Karnofsky Performance Status; LFS = Lee Fatigue Scale; m^2^ = meter squared, NK-1 = neurokinin-1; RT = radiation therapy; SCQ = Self-administered Comorbidity Questionnaire; SD = standard deviation; U = Mann-Whitney U test
