# Supplementary material for: Perturbations in common and distinct inflammatory pathways associated with morning and evening fatigue in outpatients receiving chemotherapy
Source: Cancer Med. 2022 Nov 14;12(6):7369–80. doi: 10.1002/cam4.5435 (PMC10067125; doi:10.1002/cam4.5435)
Supplement: Supplementary file 4 — Table S2. [file CAM4-12-7369-s001.docx]

Supplemental Table 2. Differences in Demographic and Clinical Characteristics Between Patients in the Microarray Sample with Low and High Morning Fatigue

| Characteristic | Low Morning Fatigue  53.3% (n=186) | High Morning Fatigue  46.7% (n=163) | Statistics |
| --- | --- | --- | --- |
|  | Mean (SD) | Mean (SD) |  |
| Age (years) | 58.2 (10.9) | 55.0 (12.5) | t = 2.56, p = 0.011 |
| Education (years) | 16.6 (3.0) | 16.2 (2.9) | t = 1.07, p = 0.287 |
| Body mass index (kg/m^2^) | 26.1 (5.8) | 27.3 (6.3) | t = -1.98, p = 0.049 |
| KPS score | 82.3 (10.9) | 76.8 (11.2) | t = 4.62, p < 0.001 |
| Number of comorbidities | 2.3 (1.3) | 2.7 (1.4) | t = -2.75, p = 0.006 |
| SCQ score | 5.1 (2.7) | 6.2 (3.2) | t = -3.59, p < 0.001 |
| AUDIT score | 3.0 (2.2) | 2.9 (2.5) | t = 0.30, p = 0.768 |
| Time since diagnosis (years) | 2.2 (3.9) | 2.3 (3.6) | U, p = 0.082 |
| Time since diagnosis (years, median) | 0.42 | 0.49 |  |
| Number of prior cancer treatments | 1.7 (1.5) | 1.9 (1.7) | t = -1.38, p = 0.170 |
| Number of metastatic sites including lymph node involvement | 1.3 (1.3) | 1.2 (1.3) | t = 0.89, p = 0.372 |
| Number of metastatic sites excluding lymph node involvement | 0.85 (1.1) | 0.82 (1.1) | t = 0.23, p = 0.822 |
| MAX2 score | 0.17 (0.08) | 0.17 (0.08) | t = -0.16, p = 0.872 |
| Hemoglobin (g/dL) | 11.8 (1.5) | 11.7 (1.3) | t = 0.88, p = 0.379 |
| Hematocrit (%) | 35.1 (4.2) | 34.8 (3.7) | t = 0.87, p = 0.383 |
|  | % (n) | % (n) |  |
| Gender  Female  Male | 77.4 (144)  22.6 (42) | 84.0 (137)  16.0 (26) | FE, p = 0.137 |
| Ethnicity  White  Black  Asian or Pacific Islander  Hispanic, Mixed, or Other | 72.6 (135)  4.8 (9)  11.8 (22)  10.8 (20) | 67.5 (110)  9.2 (15)  14.1 (23)  9.2 (15) | X^2^ = 3.29, p = 0.350 |
| Married or partnered (% yes) | 72.0 (134) | 60.7 (99) | FE, p = 0.030 |
| Lives alone (% yes) | 19.4 (36) | 20.9 (34) | FE, p = 0.789 |
| Childcare responsibilities (% yes) | 19.4 (36) | 30.1 (49) | FE, p = 0.024 |
| Care of adult responsibilities (% yes) | 10.2 (19) | 6.7 (11) | FE, p = 0.339 |
| Born prematurely (% yes) | 3.8 (7) | 6.1 (10) | FE, p = 0.329 |
| Currently employed (% yes) | 39.2 (73) | 29.4 (48) | FE, p = 0.057 |
| Income  <$30,000  $30,000 to <$70,000  $70,000 to <$100,000  ≥$100,000 | 15.1 (28)  20.4 (38)  19.4 (36)  45.2 (84) | 27.0 (44)  21.5 (35)  16.0 (26)  35.6 (58) | U, p = 0.173 |
| Specific comorbidities (% yes)  Heart disease  High blood pressure  Lung disease  Diabetes  Ulcer or stomach disease  Kidney disease  Liver disease  Anemia or blood disease  Depression  Osteoarthritis  Back pain  Rheumatoid arthritis | 5.4 (10)  29.6 (55)  11.3 (21)  7.0 (13)  3.2 (6)  0.5 (1)  7.0 (13)  10.8 (20)  15.6 (29)  12.4 (23)  20.0 (41)  3.2 (6) | 5.5 (9)  28.8 (47)  11.0 (18)  8.6 (14)  6.8 (11)  1.8 (3)  6.7 (11)  17.8 (29)  31.3 (51)  14.1 (23)  32.5 (53)  4.3 (7) | FE, p = 1.000  FE, p = 0.907  FE, p = 1.000  FE, p = 0.689  FE, p = 0.142  FE, p = 0.343  FE, p = 1.000  FE, p = 0.065  FE, p < 0.001  FE, p = 0.638  FE, p = 0.030  FE, p = 0.778 |
| Exercise on a regular basis (% yes) | 77.4 (144) | 62.6 (102) | FE, p = 0.003 |
| Smoking current or history of (% yes) | 36.6 (68) | 36.8 (60) | FE, p = 1.000 |
| Cancer diagnosis  Breast  Gastrointestinal  Gynecological  Lung | 33.3 (62)  24.7 (46)  25.8 (48)  16.1 (30) | 42.3 (69)  26.4 (43)  20.2 (33)  11.0 (18) | X^2^ = 4.76, p = 0.190 |
| Type of prior cancer treatment  No prior treatment  Only surgery, CTX, or RT  Surgery & CTX, or surgery & RT, or CTX & RT  Surgery & CTX & RT | 19.9 (37)  45.2 (84)  21.5 (40)  13.4 (25) | 16.0 (26)  44.2 (72)  20.9 (34)  19.0 (31) | X^2^ = 2.47, p = 0.481 |
| CTX cycle length  14 day cycle  21 day cycle  28 day cycle | 31.2 (58)  61.8 (115)  7.0 (13) | 36.8 (60)  54.6 (89)  8.6 (14) | X^2^ = 1.88, p = 0.391 |
| Emetogenicity of CTX  Minimal/low  Moderate  High | 21.5 (40)  61.3 (114)  17.2 (32) | 23.3 (38)  58.9 (96)  17.8 (29) | X^2^ = 0.23, p = 0.893 |
| Antiemetic regimens  None  Steroid alone or serotonin receptor antagonist alone  Serotonin receptor antagonist and steroid  NK-1 receptor antagonist and two other antiemetics | 10.2 (19)  22.6 (42)  52.7 (98)  14.5 (27) | 9.8 (16)  23.9 (39)  37.4 (61)  28.8 (47) | X^2^ = 12.92, p =0.005  NS  NS  0 > 1  0 < 1 |
| LFS morning fatigue score at enrollment | 1.5 (1.0) | 5.2 (1.4) | t = -28.48, p < 0.001 |

Abbreviations: AUDIT = Alcohol Use Disorders Identification Test; CTX = chemotherapy; dl = deciliters; FE = Fisher's exact test; g = grams; kg = kilograms; KPS = Karnofsky Performance Status; LFS = Lee Fatigue Scale; m^2^ = meter squared, NK-1 = neurokinin-1; RT = radiation therapy; SCQ = Self-administered Comorbidity Questionnaire; SD = standard deviation; U = Mann-Whitney U test
