# Supplementary material for: Perturbations in common and distinct inflammatory pathways associated with morning and evening fatigue in outpatients receiving chemotherapy
Source: Cancer Med. 2022 Nov 14;12(6):7369–80. doi: 10.1002/cam4.5435 (PMC10067125; doi:10.1002/cam4.5435)
Supplement: Supplementary file 5 — Table S3. [file CAM4-12-7369-s003.docx]

Supplemental Table 3. Differences in Demographic and Clinical Characteristics Between Patients in RNA-Seq Sample with Low and High Evening Fatigue

| Characteristic | Low Evening Fatigue  45.1% (n=157) | High Evening Fatigue  54.9% (n=191) | Statistics |
| --- | --- | --- | --- |
|  | Mean (SD) | Mean (SD) |  |
| Age (years) | 59.1 (12.1) | 54.8 (12.4) | t = 3.26, p = 0.001 |
| Education (years) | 16.0 (3.2) | 16.1 (3.0) | t = -0.29, p = 0.773 |
| Body mass index (kg/m^2^) | 26.2 (4.7) | 25.9 (5.9) | t = 0.61, p = 0.545 |
| KPS score | 80.6 (12.8) | 75.2 (11.5) | t = 4.11, p < 0.001 |
| Number of comorbidities | 2.6 (1.4) | 2.5 (1.6) | t = 0.19, p = 0.848 |
| SCQ score | 5.6 (3.2) | 6.0 (3.8) | t = -1.05, p = 0.296 |
| AUDIT score | 2.9 (2.1) | 2.7 (2.0) | t = 0.59, p = 0.557 |
| Time since diagnosis (years) | 1.6 (3.0) | 1.8 (3.2) | U, p = 0.660 |
| Time since diagnosis (years, median) | 0.47 | 0.42 |  |
| Number of prior cancer treatments | 1.4 (1.4) | 1.6 (1.4) | t = -1.52, p = 0.129 |
| Number of metastatic sites including lymph node involvement | 1.3 (1.2) | 1.1 (1.2) | t = 1.30, p = 0.196 |
| Number of metastatic sites excluding lymph node involvement | 0.87 (1.0) | 0.65 (1.0) | t = 1.93, p = 0.055 |
| MAX2 score | 0.17 (0.08) | 0.19 (0.08) | t = -1.46, p = 0.146 |
| Hemoglobin (g/dL) | 11.6 (1.5) | 11.4 (1.3) | t = 1.09, p = 0.277 |
| Hematocrit (%) | 34.7 (4.3) | 34.2 (3.8) | t = 1.10, p = 0.270 |
| LFS Evening Fatigue score at enrollment | 3.6 (1.4) | 7.0 (1.1) | t = -24.39, p < 0.001 |
| LFS Morning Fatigue score at enrollment | 2.3 (1.8) | 4.7 (2.3) | t = -10.46, p < 0.001 |
|  | % (n) | % (n) |  |
| Gender  Female  Male | 66.9 (105)  33.1 (52) | 87.4 (167)  12.6 (24) | FE, p < 0.001 |
| Ethnicity  White  Black  Asian or Pacific Islander  Hispanic mixed or other | 60.5 (95)  8.3 (13)  21.0 (33)  10.2 (16) | 66.0 (126)  7.3 (14)  13.1 (25)  13.6 (26) | X^2^ = 4.59, p = 0.204 |
| Married or partnered (% yes) | 63.7 (100) | 59.2 (113) | FE, p = 0.439 |
| Lives alone (% yes) | 18.5 (29) | 27.2 (52) | FE, p = 0.057 |
| Childcare responsibilities (% yes) | 17.2 (27) | 24.1 (46) | FE, p = 0.145 |
| Care of adult responsibilities (% yes) | 8.3 (13) | 6.8 (13) | FE, p = 0.684 |
| Born prematurely (% yes) | 1.9 (3) | 6.8 (13) | FE, p = 0.038 |
| Currently employed (% yes) | 29.3 (46) | 34.0 (65) | FE, p = 0.358 |
| Income  <$30,000  $30,000 to <$70,000  $70,000 to <$100,000  ≥$100,000 | 19.1 (30)  23.6 (37)  21.7 (34)  35.7 (56) | 22.5 (43)  17.8 (34)  19.9 (38)  39.8 (76) | U, p = 0.741 |
| Specific comorbidities (% yes)  Heart disease  High blood pressure  Lung disease  Diabetes  Ulcer or stomach disease  Kidney disease  Liver disease  Anemia or blood disease  Depression  Osteoarthritis  Back pain  Rheumatoid arthritis | 7.0 (11)  39.5 (62)  10.8 (17)  12.1 (19)  5.7 (9)  0.6 (1)  8.9 (14)  8.3 (13)  15.3 (24)  12.7 (20)  31.8 (50)  4.5 (7) | 6.3 (12)  28.3 (54)  8.9 (17)  12.0 (23)  3.7 (7)  1.6 (3)  4.7 (9)  11.5 (22)  26.2 (50)  13.1 (25)  33.5 (64)  4.2 (8) | FE, p = 0.831  FE, p = 0.030  FE, p = 0.589  FE, p = 1.000  FE, p = 0.443  FE, p = 0.630  FE, p = 0.132  FE, p = 0.373  FE, p = 0.017  FE, p = 1.000  FE, p = 0.819  FE, p = 1.000 |
| Exercise on a regular basis (% yes) | 69.4 (109) | 66.5 (127) | FE, p = 0.567 |
| Smoking current or history of (% yes) | 34.4 (54) | 35.6 (68) | FE, p = 0.822 |
| Cancer diagnosis  Breast  Gastrointestinal  Gynecological  Lung | 31.2 (49)  44.0 (69)  13.4 (21)  11.5 (18) | 46.6 (89)  26.2 (50)  17.8 (34)  9.4 (18) | X^2^ = 14.52, p = 0.002  0 < 1  0 > 1  NS  NS |
| Type of prior cancer treatment  No prior treatment  Only surgery, CTX, or RT  Surgery & CTX, or surgery & RT, or CTX & RT  Surgery & CTX & RT | 35.0 (55)  36.3 (57)  18.5 (29)  10.2 (16) | 22.0 (42)  46.6 (89)  17.8 (34)  13.6 (26) | X^2^ = 8.29, p = 0.040  0 > 1  NS  NS  NS |
| CTX cycle length  14 day cycle  21 day cycle  28 day cycle | 43.9 (69)  48.4 (76)  7.6 (12) | 49.7 (95)  41.9 (80)  8.4 (16) | U, p = 0.377 |
| Emetogenicity of CTX  Minimal/low  Moderate  High | 14.0 (22)  71.3 (112)  14.6 (23) | 17.8 (34)  57.1 (109)  25.1 (48) | U, p = 0.278 |
| Antiemetic regimens  None  Steroid alone or serotonin receptor antagonist alone  Serotonin receptor antagonist and steroid  NK-1 receptor antagonist and two other antiemetics | 3.8 (6)  17.8 (28)  56.7 (89)  21.7 (34) | 6.3 (12)  17.3 (33)  42.9 (82)  33.5 (64) | X^2^ = 8.64, p = 0.034  NS  NS  0 > 1  0 < 1 |

Abbreviations: AUDIT = Alcohol Use Disorders Identification Test; CTX = chemotherapy; FE = Fisher's exact test; kg = kilograms; KPS = Karnofsky Performance Status; LFS = Lee Fatigue Scale; m^2^ = meter squared, NK-1 = neurokinin-1; NS = not significant; RT = radiation therapy; SCQ = Self-administered Comorbidity Questionnaire; SD = standard deviation; U = Mann-Whitney U test; X^2^ = Chi-square
