# Supplementary material for: Perturbations in common and distinct inflammatory pathways associated with morning and evening fatigue in outpatients receiving chemotherapy
Source: Cancer Med. 2022 Nov 14;12(6):7369–80. doi: 10.1002/cam4.5435 (PMC10067125; doi:10.1002/cam4.5435)
Supplement: Supplementary file 6 — Table S4. [file CAM4-12-7369-s006.docx]

Supplemental Table 4. Differences in Demographic and Clinical Characteristics Between Patients in the Microarray Sample with Low and High Evening Fatigue

| Characteristic | Low Evening Fatigue  47.7% (n=166) | High Evening Fatigue  52.3% (n=182) | Statistics |
| --- | --- | --- | --- |
|  | Mean (SD) | Mean (SD) |  |
| Age (years) | 57.4 (12.4) | 55.8 (11.2) | t = 1.30, p = 0.195 |
| Education (years) | 16.1 (2.9) | 16.6 (3.0) | t = -1.59, p = 0.112 |
| Body mass index (kg/m^2^) | 26.0 (5.0) | 27.3 (6.8) | t = -2.13, p = 0.034 |
| KPS score | 81.9 (11.6) | 77.7 (10.8) | t = 3.49, p < 0.001 |
| Number of comorbidities | 2.5 (1.4) | 2.4 (1.4) | t = 0.21, p = 0.838 |
| SCQ score | 5.4 (2.9) | 5.8 (3.1) | t = -1.01, p = 0.316 |
| AUDIT score | 2.8 (1.8) | 3.2 (2.7) | t = -1.62, p = 0.107 |
| Time since diagnosis (years) | 2.0 (3.7) | 2.3 (3.8) | U, p = 0.527 |
| Time since diagnosis (years, median) | 0.43 | 0.45 |  |
| Number of prior cancer treatments | 1.8 (1.6) | 1.8 (1.6) | t = -0.41, p = 0.679 |
| Number of metastatic sites including lymph node involvement | 1.2 (1.2) | 1.3 (1.4) | t = -1.12, p = 0.264 |
| Number of metastatic sites excluding lymph node involvement | 0.72 (1.0) | 0.92 (1.2) | t = -1.61, p = 0.107 |
| MAX2 score | 0.17 (0.08) | 0.17 (0.08) | t = -0.32, p = 0.753 |
| Hemoglobin (g/dL) | 11.8 (1.4) | 11.7 (1.3) | t = 0.97, p = 0.335 |
| Hematocrit (%) | 35.1 (4.1) | 34.8 (3.8) | t = 0.58, p = 0.565 |
| LFS Evening Fatigue score at enrollment | 3.8 (1.4) | 7.1 (1.1) | t = -25.01, p < 0.001 |
| LFS Morning Fatigue score at enrollment | 2.5 (1.8) | 3.9 (2.3) | t = -6.15, p <0.001 |
|  | % (n) | % (n) |  |
| Gender  Female  Male | 77.7 (129)  22.3 (37) | 83.5 (152)  16.5 (30) | FE, p = 0.177 |
| Ethnicity  White  Black  Asian or Pacific Islander  Hispanic, Mixed, or Other | 68.1 (113)  6.6 (11)  14.5 (24)  10.8 (18) | 72.0 (131)  6.6 (12)  11.5 (21)  9.9 (18) | X^2^ = 0.84, p = 0.841 |
| Married or partnered (% yes) | 65.7 (109) | 68.1 (124) | FE, p = 0.649 |
| Lives alone (% yes) | 21.1 (35) | 18.7 (34) | FE, p = 0.593 |
| Childcare responsibilities (% yes) | 18.7 (31) | 30.2 (55) | FE, p = 0.013 |
| Care of adult responsibilities (% yes) | 8.4 (14) | 7.7 (14) | FE, p = 0.845 |
| Born prematurely (% yes) | 3.0 (5) | 6.6 (12) | FE, p = 0.141 |
| Currently employed (% yes) | 30.7 (51) | 36.8 (67) | FE, p = 0.258 |
| Income  <$30,000  $30,000 to <$70,000  $70,000 to <$100,000  ≥$100,000 | 20.5 (34)  23.5 (39)  18.1 (30)  38.0 (63) | 20.3 (37)  19.2 (35)  17.0 (31)  43.4 (79) | U, p = 0.402 |
| Specific comorbidities (% yes)  Heart disease  High blood pressure  Lung disease  Diabetes  Ulcer or stomach disease  Kidney disease  Liver disease  Anemia or blood disease  Depression  Osteoarthritis  Back pain  Rheumatoid arthritis | 7.2 (12)  30.7 (51)  13.3 (22)  7.8 (13)  4.2 (7)  0.6 (1)  4.2 (7)  12.7 (21)  20.5 (34)  13.3 (22)  28.9 (48)  4.2 (7) | 3.8 (7)  26.9 (49)  9.3 (17)  7.7 (14)  5.5 (10)  1.6 (3)  8.2 (15)  14.3 (26)  25.3 (46)  13.7 (25)  25.3 (46)  3.3 (6) | FE, p = 0.237  FE, p = 0.477  FE, p = 0.308  FE, p = 1.000  FE, p = 0.627  FE, p = 0.624  FE, p = 0.185  FE, p = 0.754  FE, p = 0.310  FE, p = 1.000  FE, p = 0.470  FE, p = 0.780 |
| Exercise on a regular basis (% yes) | 72.3 (120) | 69.2 (126) | FE, p = 0.557 |
| Smoking current or history of (% yes) | 34.9 (58) | 38.5 (70) | FE, p = 0.507 |
| Cancer diagnosis  Breast  Gastrointestinal  Gynecological  Lung | 36.7 (61)  26.5 (44)  20.5 (34)  16.3 (27) | 39.0 (71)  23.6 (43)  25.8 (47)  11.5 (21) | X^2^ = 2.88, p = 0.411 |
| Type of prior cancer treatment  No prior treatment  Only surgery, CTX, or RT  Surgery & CTX, or surgery & RT, or CTX & RT  Surgery & CTX & RT | 21.7 (36)  40.4 (67)  25.3 (42)  12.7 (21) | 15.9 (29)  48.4 (88)  17.0 (31)  18.7 (34) | X^2^ = 7.61, p = 0.055 |
| CTX cycle length  14 day cycle  21 day cycle  28 day cycle | 37.3 (62)  56.0 (93)  6.6 (11) | 31.3 (57)  61.0 (111)  7.7 (14) | U, p = 0.244 |
| Emetogenicity of CTX  Minimal/low  Moderate  High | 25.9 (43)  57.8 (96)  16.3 (27) | 18.7 (34)  61.5 (112)  19.8 (36) | U, p = 0.112 |
| Antiemetic regimens  None  Steroid alone or serotonin receptor antagonist alone  Serotonin receptor antagonist and steroid  NK-1 receptor antagonist and two other antiemetics | 7.2 (12)  25.3 (42)  50.0 (83)  17.5 (29) | 12.1 (22)  21.4 (39)  41.2 (75)  25.3 (46) | X^2^ = 6.59, p = 0.086 |

Abbreviations: AUDIT = Alcohol Use Disorders Identification Test; CTX = chemotherapy; FE = Fisher's exact test; kg = kilograms; KPS = Karnofsky Performance Status; LFS = Lee Fatigue Scale; m^2^ = meter squared; NK-1 = neurokinin-1; RT = radiation therapy; SCQ = Self-administered Comorbidity Questionnaire; SD = standard deviation; U = Mann-Whitney U test; X^2^ = Chi-square
